# Mapping Nanocellulose- and Alginate-Based Photosynthetic Cell Factory Scaffolds: Interlinking Porosity, Wet Strength, and Gas Exchange

**DOI:** 10.1021/acs.biomac.3c00261

**Published:** 2023-06-29

**Authors:** Tuukka Levä, Ville Rissanen, Lauri Nikkanen, Vilja Siitonen, Maria Heilala, Josphat Phiri, Thaddeus C. Maloney, Sergey Kosourov, Yagut Allahverdiyeva, Mikko Mäkelä, Tekla Tammelin

**Affiliations:** † VTT Technical Research Centre of Finland Ltd., VTT, P.O. Box 1000, FI-02044 Espoo, Finland; ‡ Molecular Plant Biology, Department of Life Technologies, University of Turku, FI-20014 Turku, Finland; § Department of Applied Physics, Aalto University, FI-00076 Espoo, Finland; ∥ Department of Bioproducts and Biosystems, Aalto University, FI-00076 Espoo, Finland

## Abstract

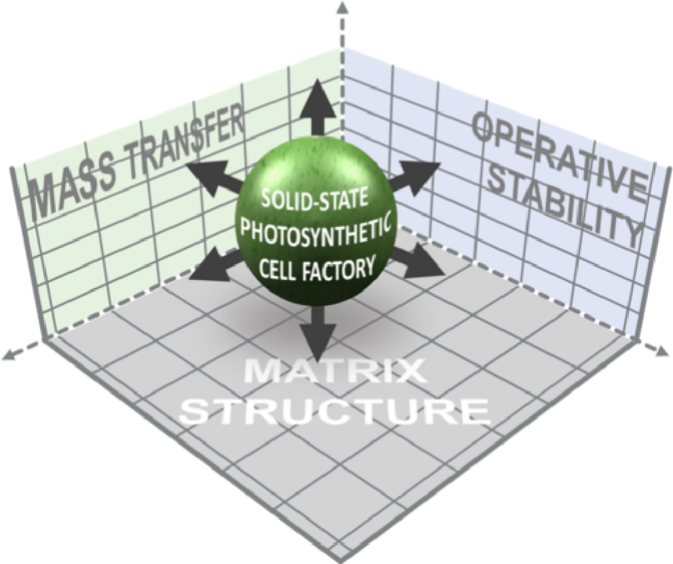

To develop efficient solid-state photosynthetic cell factories for sustainable chemical production, we present an interdisciplinary experimental toolbox to investigate and interlink the structure, operative stability, and gas transfer properties of alginate- and nanocellulose-based hydrogel matrices with entrapped wild-type *Synechocystis* PCC 6803 cyanobacteria. We created a rheological map based on the mechanical performance of the hydrogel matrices. The results highlighted the importance of Ca^2+^-cross-linking and showed that nanocellulose matrices possess higher yield properties, and alginate matrices possess higher rest properties. We observed higher porosity for nanocellulose-based matrices in a water-swollen state via calorimetric thermoporosimetry and scanning electron microscopy imaging. Finally, by pioneering a gas flux analysis via membrane-inlet mass spectrometry for entrapped cells, we observed that the porosity and rigidity of the matrices are connected to their gas exchange rates over time. Overall, these findings link the dynamic properties of the life-sustaining matrix to the performance of the immobilized cells in tailored solid-state photosynthetic cell factories.

## 1 Introduction

Whole-cell immobilization of different cell types ranging from heterotrophic microbes and animal cells to photosynthetic organisms has been extensively studied for a variety of applications including tissue engineering,^1,2^ 3D cell culture and analysis matrices,^3−5^ wastewater purification,^6−8^ and biotransformation or production of chemicals.^9−12^ In these biohybrid platforms, the properties of both the cells and the matrix are pivotal factors affecting the operational performance of the system as a whole. Cell-laden hydrogels are most often used in the biomedical field and have generally been characterized using imaging techniques that can preserve the sample ultrastructure (e.g., scanning electron microscopy (SEM), confocal laser scanning microscopy, and atomic force microscopy (AFM)),^13^ mechanical assessments (compressive and tensile testing, rheology, swelling behavior, porosity, and gel degradation),^14,15^ and by evaluating the biological compatibility of the system (cell viability, growth, and morphology).^10,11,15^ The selection of methods is heavily dependent on the end application, which defines the operational framework and boundary conditions for the applied materials.

In this article, specifically, we investigate the development of solid-state photosynthetic cell factories (SSPCFs). SSPCFs are versatile platforms for efficient and sustainable production of targeted chemicals, where photosynthetic cells are immobilized via entrapment within a thin and transparent hydrogel matrix. From the biological perspective, various photosynthetic production hosts have been extensively studied, and their robustness and productivity have been modified and enhanced with cell and metabolic engineering.^16−20^ Both the prokaryotic cyanobacteria and the diverse group of eukaryotic microalgae include species that have been identified as potential photosynthetic cell factory hosts, with a wide product range from biofuels to specialty chemicals.^21−24^ Furthermore, many investigations on suitable immobilization mechanisms and matrix components have been conducted.^9,25−27^ From the matrix perspective, hydrogels based on cross-linked alginate have been identified as well-performing materials with sustainable natural sources and high biocompatibility.^10,28−31^ Recently, hydrogels fabricated from nanocellulosic materials, such as bacterial cellulose,^32^ carboxymethylated cellulose nanofibrils,^33^ and TEMPO-oxidized cellulose nanofibers (TCNF),^11,34^ have been demonstrated as promising alternatives for conventional matrix structures due to their availability, mechanical properties, and modifiability to create tailored structures. We have previously performed a comparison between cross-linked TCNF and alginate hydrogels with regard to their mechanical performance in submerged production conditions.^11^ However, there is still a distinct need for interdisciplinary efforts combining materials science, cell biology, and bioprocess engineering to assess how the matrix properties are linked to the overall performance of the SSCPF.

**Figure 1:**
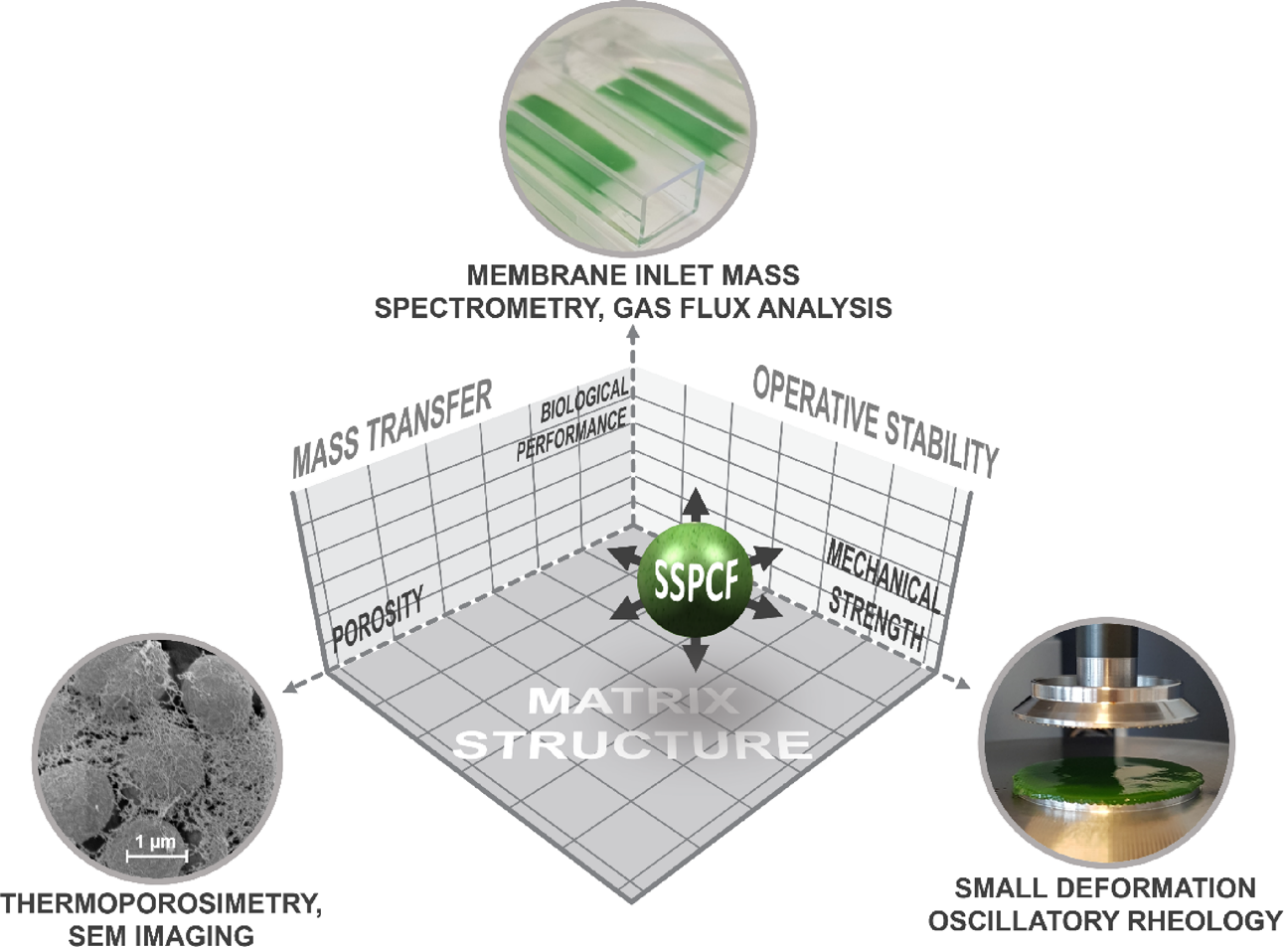
Concept of this study featuring the key dimensions and parameters for the development of solid-state photosynthetic cell factories (SSPCFs).

Here, we bring together a strong multidisciplinary expertise combining materials engineering, chemometrics, biotechnology, and photosynthesis research to form a thorough investigation of these matrix materials with many experimental methods that are novel to the SSPCF concept and its development. We present a systematic investigation of the structure, mass transfer, and operative stability of TCNF- and alginate-based SSPCF matrices and mixtures thereof (Figure 1). These materials have been investigated in detail in the context of many other application areas.^5,35−39^ However, the characterization techniques are often not directly applicable to SSPCF matrix scaffolds, where biological compatibility with the entrapped production hosts necessitates a very specific interplay between different material properties such as hydrogel strength, wet-state porosity, and gas transfer efficiency. To overcome this lack of established methodology, we identified an experimental toolbox that could characterize the relevant properties of this unique system. This toolbox was used for the mapping of the matrix materials for constructing versatile SSPCFs with tailored properties. Moreover, as cross-linking of alginate and TCNF is required to obtain self-standing matrices, we deepened the investigation of the effect of calcium-^40^ and PVA-based^41^ cross-linking approaches on the matrices.

We employed small deformation oscillatory rheology to investigate the wet strength of the matrices under shearing. We then determined the systematic variations and groupings in matrix properties with principal component analysis (PCA)^42^ and unsupervised hierarchical clustering.^43^ As a result, we were able to highlight the important rheological features governing the matrix compositions and cross-linking types. To observe the porosity of the matrices in the wet state, we used a technique called thermoporosimetry (TPM), which is based on calorimetric scanning and has been employed to calculate the pore volumes and size distributions of mesoporous materials including nanocellulose networks.^44−46^ The results from thermoporosimetry were complemented by imaging the matrix surfaces with scanning electron microscopy (SEM). Finally, we entrapped *Synechocystis* sp. PCC 6803 wild-type cyanobacterial cells in the matrices and assessed the relationship between the matrix porosity and the biological performance of the immobilized cells by following the gas transfer of the matrices. The kinetics and net gas exchange of CO_2_ and O_2_ during controlled periods of light and dark were investigated with membrane inlet mass spectrometry (MIMS), which has not been used before in the research aiming at developing SSPCFs.

We found that despite being confined to operate near the limits of our chosen techniques, we were able to observe distinctions in the initial wet strength, porosity, and gas transfer properties between TCNF- and alginate-based matrices. The mixed formulations exhibited properties mostly from the dominant component. Additionally, we identified the use of calcium ions as an effective cross-linking mechanism for both TCNF and alginate. Notably, the different interactions between the matrix components and the ions lead to diverse material behavior that can attribute to dynamic changes in matrix properties. Overall, the interdisciplinary methodological toolbox presented in this work provides a means to better understand the complex and interdependent relationship between matrix properties and SSPCF performance. This knowledge is required to develop tailorable cell immobilization matrices that can be fitted to the needs of specific production hosts and operating conditions.

## 2 Materials and Methods

### 2.1 Cyanobacterial Cells and Growth Conditions

Wild-type *Synechocystis* sp. PCC 6803 cyanobacterial cells (herein denoted as *Synechocystis*) were cultivated in the BG-11 growth medium^47^ that was buffered with 5 mM HEPES-NaOH (pH 7.5). The cell cultures were inoculated to 250 mL conical flasks in a total culture volume of 50 mL and placed on a rotary shaker (110 rpm). The suspension cultures were incubated at 23 °C under ambient CO_2_ levels and 16 h of photoperiod illuminated by fluorescent lamps (Philips Master TL5 HO 39W/865) supplying the cultivated photosynthetic cells with approximately 50 μmol photons m^–2^ s^–1^ photosynthetically active radiation (PAR). The cultures were periodically renewed in an open laminar flow cabinet (KOJAIR) after growing them for 7–14 days.

### 2.2 TEMPO-Oxidized Cellulose Nanofibers (TCNF)

TEMPO-oxidized cellulose nanofibers were manufactured from never-dried bleached softwood pulp that was obtained from a coniferous wood mixture consisting of spruce and pine. Pulping was carried out in a Finnish pulp mill, and the TEMPO-catalyzed oxidation of the pulp was conducted with alkaline hypochlorite as the primary oxidant according to the protocol reported by Saito et al.^48^ 2,2,6,6-Tetramethylpiperidine-1-oxyl (TEMPO) (Sigma-Aldrich) and 10% sodium hypochlorite (5 mmol/g pulp fiber, Sigma-Aldrich) were used in the TEMPO-catalyzed oxidation of the pulp. After pulping and oxidation, an anionic charge of 1.45–1.52 mmol/g was obtained for the oxidized pulp via a standard conductometric titration method (SCAN 65:02).^49^ The oxidized pulp was subsequently washed and passed through a microfluidizer carrying two Z-type chambers with respective diameters of 400 and 100 μm (Microfluidics Int., USA) twice at 1850 bar to fibrillate the oxidized pulp into TCNF. TCNF of ∼1 wt % with viscous gel-like characteristics and transparent optical properties was obtained. The chemical composition, morphology, and visual appearance of similarly prepared TCNF-grade used in this study were described in earlier publications.^11,34^

### 2.3 Polymers

Two polymeric components, alginate (ALG) and polyvinyl alcohol (PVA), were used in addition to TCNF in the preparation of hydrogel cell immobilization matrices. Alginic acid sodium salt from brown algae (#71238, Sigma-Aldrich) was used in this study, with a β-d-mannuronic acid (M) content of 25–35%, α-l-guluronic acid (G) content of 65–70% (M/G ratio 0.43), and 100–200 kg/mol Mw, as approximated by the manufacturer. Alginate was dissolved in Milli-Q water overnight with magnetic stirring to prepare stock solution of approximately 2 wt %. PVA (Mowiol 56-98, Mw: 195 kg/mol, DP 4300, Sigma-Aldrich), and stock solution of around 5 wt % was prepared by dissolving solid PVA crystals to Milli-Q water for approximately 1 h until fully dissolved. The water solubility of PVA was enhanced by stirring and heating the solution to >90 °C in a water bath during preparation.

### 2.4 Other Materials

Ultrapure Milli-Q water (18.2 MΩ cm) used throughout this study in the preparation of solutions and samples was obtained with a Milli-Q purification unit (QPAK 1, Millipore). Calcium chloride (50 mM) (CaCl_2_) solution was prepared by dissolving CaCl_2_ of analytical grade (99%, #C7902, Sigma-Aldrich) to Milli-Q water. A commercial Teflon (PTFE) film (Etra, Finland) was used as a scaffold during the preparation of all hydrogel films.

### 2.5 Preparation of Cell Immobilization Matrices

Seven types of different hydrogel matrices with varying cross-linkers were prepared. All matrices were prepared to an approximately final 1 wt % solid content and 1 ± 0.5 mm thickness prior to immersion in either Milli-Q water (matrices with no cells) or BG-11 growth medium (matrices with cells). Samples were let to swell until equilibrium, which resulted in the materials having slightly different solid contents during further measurements. A schematic representation of the matrix preparation and cell immobilization (Figure S1), a table containing the composition of the cell immobilization matrices before swelling (Table S1), and AFM topography and phase contrast images of Ca^2+^-cross-linked TCNF and alginate films (Figure S2) are shown in the Supporting Information.

#### 2.5.1 Preparation of Matrices with TCNF

Matrices with TCNF were prepared both with and without Ca^2+^-cross-linking. Materials with calcium cross-linking were referred to with “Ca-0.5TCNF-0.5ALG”, “Ca-0.9TCNF-0.1ALG”, “Ca-1.0TCNF”, and “Ca-1.0TCNF-0.1PVA”, where the numbers before the main matrix components, TCNF, and alginate accounted for their ratio in the final 1 wt % mixture. If present, PVA was added by an amount corresponding to 10% of the dry weight of TCNF in the mixture, which was signified with “0.1” before PVA in the name of the material. Similarly, materials without calcium cross-linking were referred to with “1.0TCNF” and “1.0TCNF-0.1PVA”. All matrices with TCNF were prepared in three main steps: (i) mixing, (ii) casting, and (iii) rewetting. During the mixing phase, matrix components were homogenized with a digital T 25 Ultra-Turrax homogenizer (IKA, Staufen, Germany) at 12,000 rpm for 3 min, after which air bubbles were removed via centrifugation at 4500*g* for 3 min. In the mixing phase, the material was prepared to contain 0.5 wt % of the main matrix components (TCNF or TCNF-alginate combinations). Then, the matrices were cast on a solid support coated with a PTFE film to a thickness of 2 mm. Casting of the matrices was followed by the primary cross-linking with Ca^2+^-ions. Thorough cross-linking of the matrix materials was induced by spraying 50 mM CaCl_2_ on the gels until wet, as described earlier.^11,29^ The gels without Ca^2+^-cross-linking were sprayed with Milli-Q water until wet. Then, the matrices were dewatered to a 1 wt % solid content in 23 °C and 50% relative humidity (RH) to enhance penetration of Ca^2+^ to the nanofibril network and to promote esterification between TCNF and PVA for additional cross-linking. After dewatering, individual samples were cut from the prepared materials. The samples with Ca^2+^-cross-linking were then submerged in 50 mM CaCl_2_ for 15–30 min to eliminate the chance of network collapse or loosening of the cross-link in freshly cut sample edges. Finally, the samples were left to swell overnight (16–24 h) before any successive measurements. Matrices without cells were swelled in Milli-Q water, whereas matrices with immobilized cells were swelled in the BG-11 growth medium.

#### 2.5.2 Preparation of the Ca^2+^-Alginate Matrix

Calcium-alginate matrices, referred to as “Ca-1.0ALG”, were otherwise treated similarly to the matrices with TCNF in the mixing phase, but they were prepared directly to a 1 wt % solid content. In the casting phase, the mixture was poured on a solid support coated with a PTFE film to a thickness of 1 mm and cross-linked by spraying 50 mM CaCl_2_ on top until wetted. The Ca^2+^-ions were let to diffuse into alginate for approximately 45 min, after which the samples were cut from the solidified gel and rewetted as described for TCNF-containing matrices.

#### 2.5.3 Cell Immobilization

Wild-type *Synechocystis* cells were immobilized to the prepared hydrogel matrices via passive gel entrapment. Prior to immobilization, photosynthetically active *Synechocystis* cells were collected by pelleting the cells by centrifugation at 10,000*g* for 15 min. The cell concentrate was diluted to have an optical density (OD_720_) of 1.0 ± 0.1. The optical density measurements were performed with an AquaPen-C AP-C 100 handheld pulse amplitude modulation (PAM) chlorophyll fluorometer (Photon Systems Instruments, Czech Republic) calibrated with the BG-11 medium. The cell suspension was then added to the hydrogel constituents in a 1:1 ratio during the mixing phase instead of water or the BG-11 medium.

### 2.6 Rheological Mapping of Matrix Materials

#### 2.6.1 Small Deformation Oscillatory Rheology

Thin-layer hydrogels with a flat design were studied with small deformation oscillation stress sweep measurements utilizing Discovery HR-2 and AR-G2 -rheometers (TA Instruments, New Castle, DE, USA). Serrated parallel plate geometry with a diameter of 40 mm was used in the measurements to avoid wall slip. The serrated baseplate of the instrument was set at 22 °C with a Peltier plate. Before measurements, the hydrogel samples were let to adjust to compression for 2 min under the measuring head. The normal force between the sample and the rheometer was followed when lowering the measuring head to the thickness of the sample. The gap was adjusted until a normal force between 0.3 and 1 N was observed. Samples with a uniform thickness of 1000 ± 500 μm were selected for further analysis, except for hydrogels without Ca^2+^-cross-linking where all samples were thicker than 1500 μm after swelling. The stress sweeps were performed in an oscillation stress range from 0.1 to 600 Pa under a constant frequency of 0.1 Hz. During the measurement, 10 points per measuring decade were taken. Controlling the rheometer and data collection was conducted with a TRIOS program (TA Instruments, New Castle, DE, USA).

**Figure 2:**
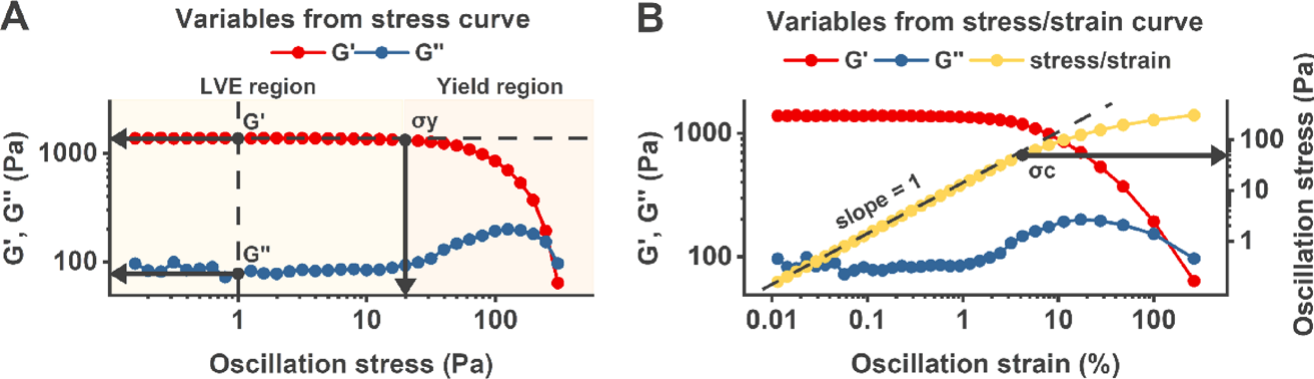
Rheological parameters used as the basis for principal component analysis and rheological comparisons. (A) Typical stress curve of the materials, where *G*′, *G*″, and tan δ (*G*″/*G*′) were determined from the linear viscoelastic (LVE) region at an oscillation stress of 1 Pa and σ_y_ from the point where *G*′ decreases 5% from the LVE region. (B) Typical stress–strain curve of the materials, where σ_c_ was determined at the point of a 15–20% decrease from the linear slope.

With small deformation oscillatory rheology, the viscoelastic properties of prepared hydrogels were interpreted in the linear viscoelastic region (LVE region) and during material yielding. The elastic and viscous components of a material were characterized by the storage and loss moduli (*G*′ and *G*″, unit Pa).^50^ The loss tangent (tan δ), or damping factor, was determined as the ratio of *G*″ to *G*′ (tan δ = *G*″/*G*′). *G*′, *G*″, and tan δ were determined from the linear viscoelastic region (LVE region) of a stress sweep curve (Figure 2A), at 1 Pa oscillation stress where *G*′ and *G*″ were independent of the applied shear stress. Material yielding properties were characterized with yield stress (σ_y_) and critical stress (σ_c_). Yield stress (σ_y_) denoted the onset of non-linear behavior in the modulus response to the applied shear stress at the limit of the LVE region. It was determined from the stress sweep curve as the point where *G*′ had decreased approximately 5% from its preceding linear value (Figure 2A). Critical stress (σ_c_) indicated the system’s transition to predominantly irreversible deformation. It was determined from the stress/strain curve (Figure 2B) by obtaining the oscillation stress value where the slope of the stress/strain curve had decreased 15–20% from the linear region, i.e., the strain was no longer linearly dependent on the stress.

#### 2.6.2 Multivariate Data Interpretation

Differences in the rheological parameters of the hydrogel matrices were determined using principal component analysis (PCA) and hierarchical clustering. The measured rheological parameters (*G*′, *G*″, tan δ, σ_y_, and σ_c_) of the samples were compiled into a 46 × 5 raw rheological data matrix (Table S2) where the samples described in Section 2.5 were given as row objects and the parameters as the corresponding columns. The data matrix was preprocessed by normalizing the columns to unit variance and zero mean to enable comparing variables given in different units. The preprocessed data were then decomposed into principal component (PC) scores and loadings according to the general PCA model^51^ (eq 1):
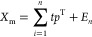
1where *X*_m_ denoted the preprocessed and mean-centered data matrix, *t* is the orthogonal score vectors, *p*^T^ is the orthonormal loading vectors, and *E_n_* is the residual matrix after the calculation of *n* components. With normalized and mean-centered data, the PC loadings were equal to the eigenvectors associated with the largest eigenvalues of the correlation matrix of *X*_m_. The number of rheologically relevant components was estimated based on the scree plot (Figure S3),^52^ and the hydrogel differences were explained by the scores of each component. The samples were then divided into distinct classes by clustering the determined PC scores. The clusters were formed by minimizing the sums of squared Euclidean distances between the sample scores and the cluster centroids using Ward’s method.^53^ The final number of clusters was determined by identifying increasing cluster distances from the resulting dendrogram.^43^ Origin 2021b (OriginLab Corporation, Northampton, MA, USA) was used to calculate and plot the outputs from multivariate data analyses.

### 2.7 Thermoporosimetry

The pore structure of the hydrogels was analyzed using the thermoporosimetry (TPM) method. The measurements were conducted on a Mettler Toledo DSC 3+ (Mettler-Toledo Intl. Inc. Instrument, USA) differential scanning calorimeter equipped with an intracooler. The samples in triplicates were hermetically sealed in 40 μL aluminum pans. The masses of the sealed crucibles were recorded before and after the measurements to ensure that there was no leakage during the measurement. After the measurements, the crucibles were punched with a needle and dried in an oven at 105 °C overnight to determine the moisture content. The temperature was first brought to −50 °C at 20 K min^–1^ to crystallize all the freezable water in the samples. The temperature was then increased to −0.2 °C and held constant until the melting transition was completed, i.e., until all the water in the small capillaries melt. This step is essential to prevent supercooling during the subsequent recrystallization step. The temperature was then decreased at 2 K min^–1^ to −50 °C. The relationship between the pore diameter (*D*) and the melting temperature depression was described by the Gibbs–Thomson equation, and the diameter was calculated from the modified version according to Maloney.^45^ The pore volume distributions in this study included only the fraction of water in the sample that freezes. The interfacial water layer, which does not freeze, represented only a small fraction of the overall pore volume and was not included in the analysis. The pore volumes were expressed in milliliters of water/g solids. It was assumed that the specific gravity of water is 1, and deviations from this value due to temperature effects were ignored.

### 2.8 SEM Imaging

Scanning electron microscopy (SEM) was used to compare the network morphology in different matrices. Hydrogel samples were cut into 5 mm × 5 mm pieces, and water was removed through solvent exchange with ascending series of 30 × 100, 50 × 100, 70 × 100, 90 × 100, and 6 × 100% ethanol. The sample was kept in each solution for 15 min, except for the first five 100% ethanol steps that were 1 h each and the final exchange that occurred overnight. Dehydrated samples were placed in a Bal-Tec CPD 030 critical point dryer (Bal-Tec, Liechtenstein) where ethanol was substituted for liquid CO_2_ over several fill-purge cycles at 5–10 °C. Finally, the temperature was increased to 40 °C to raise the pressure to approximately 90 bar and bring CO_2_ above its supercritical point. Gaseous CO_2_ was released, and the dried sample was mounted onto an SEM stub with adhesive carbon tape. The sample was immediately coated with 4 nm iridium in a Leica EM ACE600 sputter coater (Leica Microsystems, Germany). SEM images were acquired using a scanning electron microscope ZEISS Sigma VP with Gemini column (ZEISS, Germany) at 1 kV acceleration voltage.

### 2.9 Gas Exchange Analysis from Immobilized Cells

An in-house built membrane inlet mass spectrometry (MIMS) system^54^ was used to monitor the in vivo O_2_ and CO_2_ fluxes from *Synechocystis* cells entrapped in hydrogel matrices. In the technique, a semi-permeable membrane in the MIMS chamber separates the sample from a mass spectrometer, where ionized gases are detected and distinguished based on their mass/charge (*m*/*z*) ratio.^55^ A 1 cm × 1 cm piece of the hydrogel matrix was placed in the MIMS sample chamber with 1 mL of the BG-11 medium (pH 7.5). HCO_3_^–^ was added to a final concentration of 1.5 mM to ensure sufficient carbon supply for photosynthesis during the experiments. The gas fluxes were then monitored during a 5 min dark adaptation, a 5 min illumination period of 500 μmol photons m^–2^ s^–1^, and a 5 min post-illumination dark period. Gas exchange rates were calculated based on the chlorophyll content in the hydrogel matrix pieces according to the protocol reported by Beckmann et al.^56^ Maximal O_2_ (*m*/*z* 32) evolution and CO_2_ (*m*/*z* 44) fixation rates were calculated as average rates for 3–5 min after the onset of illumination and dark respiration rates as average rates during pre-illumination darkness. Net O_2_ evolution was calculated as maximal O_2_ evolution – dark respiration.

### 2.10 Determination of the Chlorophyll Content in Hydrogel Matrices

The chlorophyll *a* (Chl) concentration was determined by incubating the film pieces (1 cm × 1 cm) in 3 mL of 90% (v/v) methanol at +4 °C overnight in the dark. Prior to measurements, the samples were centrifuged with a tabletop centrifuge with full speed for 1 min. Absorbance was measured from the supernatant at 665 and 730 nm using a UV-1800 spectrophotometer (Shimadzu, Japan), and the 730 nm absorbance values were subtracted from the 665 readings and multiplied by 12.7 to get the chlorophyll concentration.^57^

## 3 Results and Discussion

### 3.1 Mapping the Rheological Properties of Hydrogel Immobilization Matrices

We studied the rheological properties of water-swollen hydrogel cell immobilization matrices with different material constituents and cross-linkers using small deformation oscillatory stress sweeps. The aim was to determine systematic differences and potential groupings within the materials based on their wet strength and visualize these groups as a “rheological map”. Seven different hydrogel matrices were prepared in water and without immobilized cells using either alginate, TCNF, or their mixtures. The cross-linking effect of Ca^2+^-ions was investigated with all matrices, and PVA was used for TCNF-based matrices.

**Figure 3:**
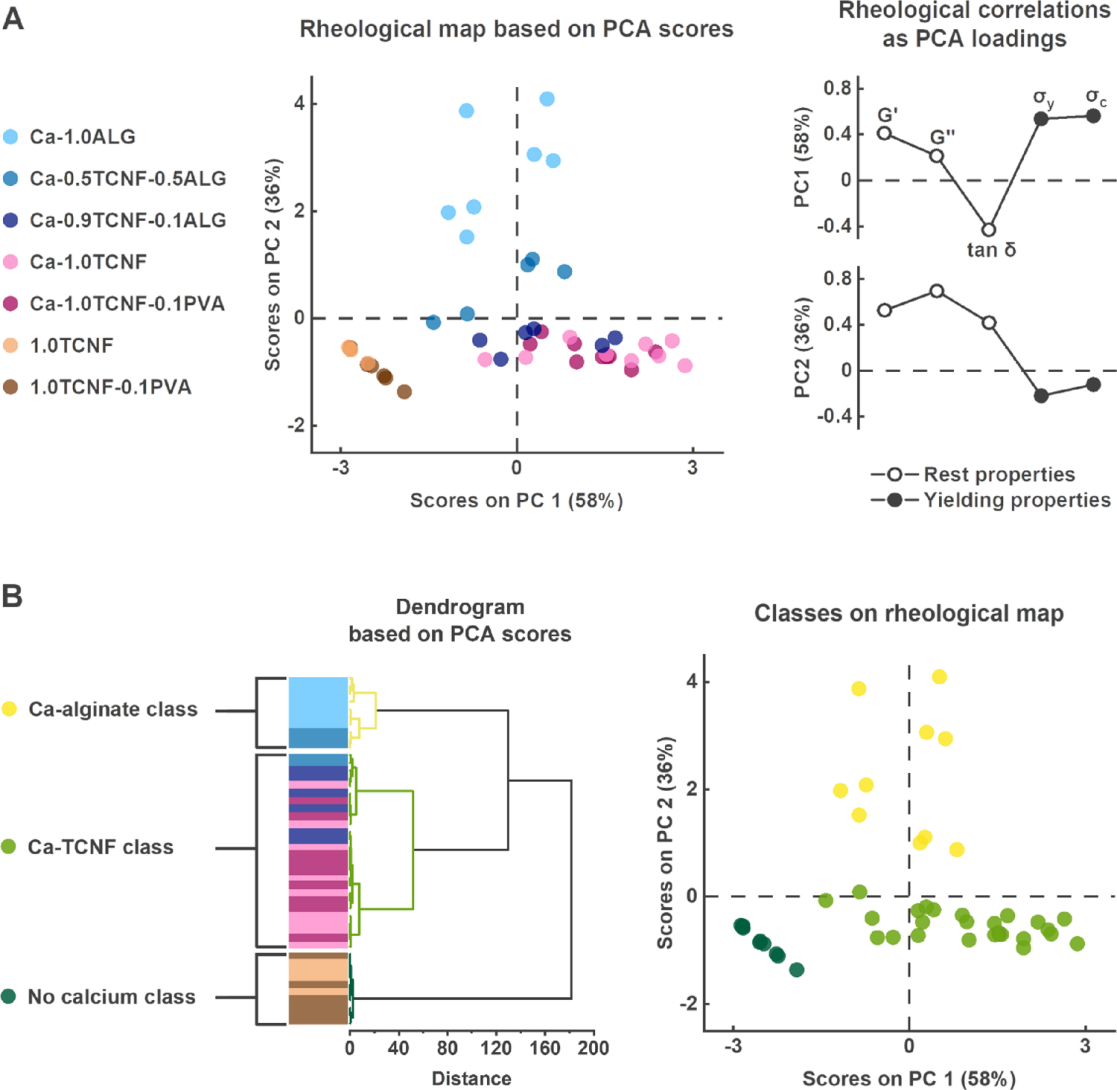
Rheological map and hierarchical clustering of the matrix properties. (A) Rheological map based on the determined PCA scores (left) and loadings (right). (B) Unsupervised hierarchical clustering of matrix materials presented as a dendrogram (left). Results from the clustering are illustrated on the rheological map according to their assigned classes (right).

The PCA results described the rheological features of the matrices and are shown in Figure 3A. The score plot illustrates the differences within the samples, and the corresponding loadings provide their interpretation based on the determined rheological parameters. In PCA, positive score values have a positive correlation with positive loadings and vice versa. Because the PCs reduce multivariate measurement space to a few interpretable dimensions, they can be used to explore and identify important systematic variations in the data and are useful for identifying sample groupings. As shown in Figure 3A, the first two PCs explained 94% of the variation in the preprocessed data, providing a meaningful interpretation of the rheological properties of the hydrogels. The later PCs did not provide any further information and as suggested by the scree plot (Figure S3) were most likely attributed to measurement noise.

The PCA illustrates that the TCNF-based matrices with Ca^2+^-cross-linking (Ca-0.9TCNF-0.1ALG, Ca-1.0TCNF, and Ca-1.0TCNF-0.1PVA) had high scores on PC1 and low scores on PC2. Furthermore, the TCNF-based matrices without Ca^2+^-cross-linking (1.0TCNF and 1.0TCNF-0.1PVA) had comparable scores on PC2 but negative scores on PC1. PC1 explained 58% of the data variation, and as seen from the loadings, higher scores were associated with higher *G*′, *G*″, σ_y_, and σ_c_ and lower tan δ values compared to other matrices. Thus, PC1 scores can be interpreted as the yield behavior and wet strength of the hydrogels, and the loadings generally suggested that elastic behavior at rest (i.e., input energy stored within the material structure) led to later yielding and progression to viscous behavior.

The matrices with a high alginate content (Ca-0.5TCNF-0.5ALG and Ca-1.0ALG) were clearly different from the TCNF-based matrices, possessing lower PC1 and higher PC2 scores. PC2 explained 36% of the remaining data variation, and the corresponding loadings indicated that high scores were associated with higher *G*′, *G*″, and tan δ values and, to some extent, lower σ_y_ and σ_c_ values compared to other matrices. This can be seen to reflect on the differences in the chemical composition of the hydrogels and the cross-linking effect of calcium ions before yielding. PC2 also suggested that increasing the *G*″/*G*′ ratio, i.e., higher tan δ values, led to material yielding in lower oscillation stresses during shearing.

The PCA sample scores were clustered into distinct classes to assess if the rheological differences were indeed related to specific matrix formulations. The scores of the first two PCs were used, and the clusters were determined with Ward’s method.^53^ The results are shown in Figure 3B. Three final hydrogel classes were chosen based on increasing cluster distances shown in the dendrogram. The first class included all Ca-1.0ALG samples and three Ca-0.5TCNF-0.5ALG samples (10 samples) with the highest rest behavior properties and was therefore denoted as the “Ca-alginate class”. The second class included two of the remaining Ca-0.5TCNF-0.5ALG samples and all TCNF-based matrices with Ca^2+^-cross-linking (26 samples) and was named the “Ca-TCNF class”. Finally, the remaining TCNF-based matrices without Ca^2+^-cross-linking (10 samples) formed the “no calcium class”. Notably, the Ca-TCNF class bore more resemblance to the Ca-alginate class than to the no calcium class. Thus, it is clear that ionic cross-linking in particular affected the rheological behavior of TCNF-based hydrogel matrices.

The average rheological characteristics of each class are shown in Table 1. With *G*′ values of over 1000 Pa, both Ca-alginate and Ca-TCNF classes showed predominantly elastic behavior and hence good capability of storing energy in their internal chemical and physical bonds in the LVE region. The matrices in the Ca-alginate class had especially high *G*′, which lends rigidity to the material structures at rest. However, they started yielding and progressed into predominantly viscous deformation at oscillation stress values 2 times lower than the Ca-TCNF class.

**Table 1:** Average (Mean ± SD) Rheological Properties of the Determined Hydrogel Classes^a^

	rest properties			yield properties	
class	*G*′ (Pa)	*G*″ (Pa)	tan δ (*G*″/*G*′)	σ_y_ (Pa)	σ_c_ (Pa)
Ca-alginate class	2400 ± 660	280 ± 86	0.12 ± 0.014	18 ± 14	52 ± 20
Ca-TCNF class	1500 ± 340	100 ± 17	0.071 ± 0.013	43 ± 18	100 ± 42
no calcium class	18 ± 4.0	2.0 ± 0.20	0.11 ± 0.016	2.0 ± 0.50	6.0 ± 3.0

a *G*′ = storage modulus, *G*″ = loss modulus, tan δ = the loss tangent (i.e., damping factor), σ_y_ = yield stress, σ_c_ = critical stress. Variables are defined in Section 2.6. The values are rounded to two significant figures.

**Figure 4:**
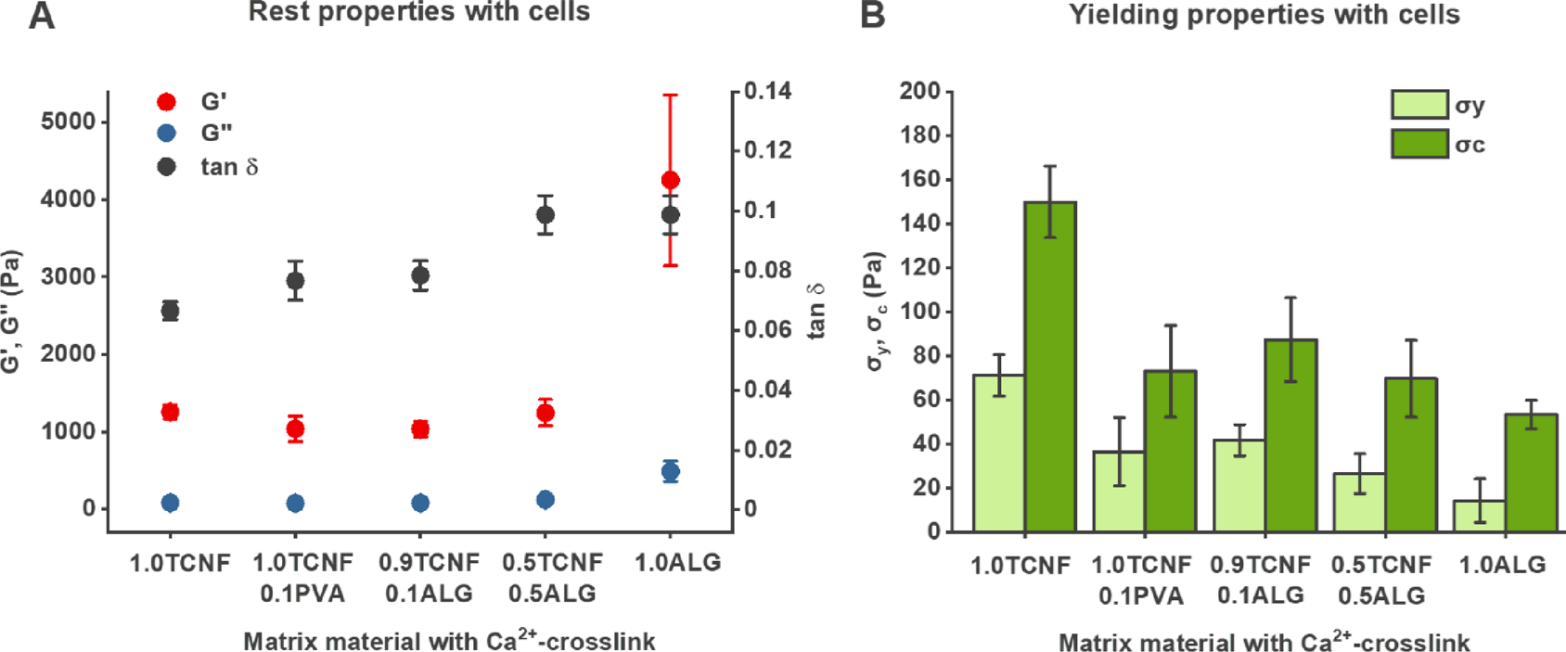
Rheological properties of Ca^2+^-cross-linked matrices from Ca-TCNF and Ca-alginate classes with immobilized *Synechocystis* cells. (A) Materials at rest, showing *G*′, *G*″, and tan δ. (B) Materials during yielding, showing yield stress (σ_y_) and critical stress (σ_c_). Sample size: *n* = 4 for Ca-1.0TCNF and Ca-1.0ALG, *n* = 6 for Ca-0.9TCNF0.1ALG and Ca-0.5TCNF0.5ALG, and *n* = 11 for Ca-1.0TCNF0.1PVA.

Ca^2+^-cross-linked matrices in the Ca-alginate and Ca-TCNF classes were chosen for further rheological evaluations with immobilized *Synechocystis* cells. The results at rest and during yielding are shown in Figure 4.

With the cells included, the *G*′ and *G*″ values of all TCNF-containing matrices fluctuated roughly around 1000 and 100 Pa, respectively. For Ca-1.0ALG matrices, the values were much higher: *G*′ varied between 3000 and 5000 Pa and *G*″ at 300 Pa. The tan δ values increased with the proportional alginate content, but here, Ca-0.5TCNF0.5ALG was comparable to Ca-1.0ALG, suggesting that it shares properties with both matrix types. As with the PCA-assisted mapping, the results reflected the properties of the dominating matrix component, i.e., the energy lost to dissipation (viscous behavior) versus stored in the material (elastic behavior) increased in the matrices with higher alginate contents.

The ratio of elastic to viscous behavior of the matrices at rest seems also to correlate with the yield properties shown in Figure 4B. Ca-1.0TCNF had the highest and Ca-1.0ALG the lowest yield properties with the mixed matrix formulations in between. However, here, the difference between Ca-1.0TCNF and the other TCNF-containing matrices was much larger. Moreover, Ca-1.0TCNF-0.1PVA and Ca-0.9TCNF-0.1ALG had very similar rheological properties overall, which suggests that a small addition of either polymer softens the TCNF matrix in a comparable manner. The ratio of yield stress to critical stress was consistent throughout the different matrices, except for Ca-1.0ALG, which began to yield at much lower critical stress than any other material. Finally, the addition of cells caused minor changes in the rest properties of all matrices (Figure S4), with the tan δ values of TCNF-containing matrices increasing slightly. Yield values, however, fall well within the error margins for all samples.

Determining the wet mechanical properties of the hydrogel matrices subject of this investigation is challenging due to the unique nature of their structures. The samples are not strong enough for conventional tensile testing, and results from compression measurements do not represent real conditions in photobioreactors very well. In contrast, shear stresses are reported to have a particularly profound effect in these systems.^58−60^ The effect of shear stress on materials can be studied with small and large deformation oscillatory rheology. The former gives accurate information in the LVE region and at the onset of nonlinear (yielding) behavior of gel-like materials,^61^ while the latter provides insight to their flow and fracture properties.^62^ This makes small deformation rheology more favorable for our investigations as we are not interested in the flow properties of the materials, and the formation of fractures can be deemed more indicative of heterogeneous structural weaknesses in a gel matrix in comparison to yield properties.^63^

Nonetheless, we have found that the matrices investigated here are inherently heterogeneous and also much stiffer than typical samples measured with small deformation oscillatory rheology, which leads to high variance, especially after the onset of nonlinear behavior. This is largely due to the increased likelihood of slipping as the shape and roughness of the sample surfaces are difficult to control, leading to uneven contact in the plate–plate measurement setup. The use of a serrated plate is known to alleviate these difficulties,^64^ and we have improved the reproducibility through a systematic approach in sample preparation, validation, and measurement protocols presented here. For further in-depth mechanical investigations of self-standing hydrogels, we also recommend using localized stress sweeps with added measurement points in the predetermined yield region of the materials to improve the measurement sensitivity in the region.

Here, we studied the rheological properties of the samples using a dataset with higher number of replicates than in our previous study^11^ and identified clear trends across different matrix compositions based on PCA and clustering. The results were presented as a rheological map, which successfully differentiated the materials into distinct classes (Figure 3). Our approach is novel in studying the rheology of self-standing matrices and offers two main advantages. First, PCA rotated the original measurement axes toward the direction of maximum variation and captured the most important correlations across the rheological measurements in the PCA loadings. These loadings provide an interpretation of the dominant rheological features of the matrices by separating non-systematic variations generated by empirical uncertainties. Second, the corresponding PCA scores enabled us to visualize the most important differences across the samples in just a few interpretable dimensions. We further clustered these scores into distinct classes, which describe the main matrix types based on their determined rheological characteristics.

According to the results, the hydrogel matrices yielded at higher oscillation shear stresses (high σ_y_ and σ_c_) with increasing TCNF concentration, although they possessed weaker rest behavior properties than the matrices with high alginate concentrations. Notably, without the ionic cross-linking, the TCNF-based matrices had weak rest and yield properties and were not self-standing, even with the incorporation of PVA as an additional cross-linker. With increasing alginate concentration, the Ca^2+^-cross-linked matrices became more rigid with high elastic capacity at rest, but due to their simultaneous susceptibility to energy dissipation, they yielded in low oscillation stresses.

The dual nature of high *G*′ and low yield values for alginate-based matrices is likely a result from intrinsic attributes of the chemistry behind the gelation of alginate. Alginate forms gels with divalent cations through development of specific coordination complexes that are often explained via the “egg-box model”, proposed by Grant et al.^65^ Although the egg-box model has undergone revisions during the 21st century,^66,67^ it serves as the principally accepted mechanism and foundation for the multistep gelation process of alginate.^68,69^ The gelation is understood to be a synergistic effect of intramolecular stereochemistry, polyelectrolyte effect, and sequential association between alginate chains: the Ca^2+^-ions bond with the deprotonated carboxylate anions present in G residues in a critical nucleation step followed by the formation of egg-box dimers between successive G units (GG-blocks) and their lateral association to multimers.^68,69^

The ionic cross-links in alginate are reversed if stronger ligands than those participating in the gelation process are added to the system. This causes an alginate gel to dissolve into a viscous liquid. Similarly, if high enough input stress is introduced to the gel, then the egg-box structures begin to break, or “unzip”, and irreversible material deformation via fracture initiation and propagation takes place.^70^ Thus, alginate-based immobilization matrices are inherently susceptible to both chemical and mechanical stresses. Furthermore, alginate contains MM-blocks and mixed MG-blocks in ratios depending on the source of the alginate. The blocks that contain mannuronic acid subunits interact weakly with Ca^2+^-ions in comparison to GG-blocks, effectively reducing the active surfaces required for gelating.^67^ Overall, the ion-coordination complexes produce elastic capacity in alginate matrices, leading to a more rigid material at rest, but the reversibility and specificity of the bonds along with the viscous nature of alginate solutions make alginate-based gel matrices susceptible to breaking under shear stress.

Compared to alginate, TCNF are larger structural units consisting of numerous cellulose polymer chains with carboxylic groups only on the C6 positions. Thus, the interfibril Ca^2+^-cross-links of TCNF via Ca^2+^-carboxylate complexes^71^ must bind much larger structures together than the interchain Ca^2+^-cross-links between individual alginate polymer chains.^5,67,69^ However, due to the high aspect ratio of TCNF, they form a highly viscous gel-like material at a low solid content even without cross-linking. Upon addition of divalent or multivalent cations, the interfibril repulsive forces caused by the carboxylate surfaces are effectively screened^71^ and the formation of Ca^2+^-carboxylate complexes, though weaker than in the egg-box structures of alginate, increases their overall yield and critical stresses to higher values than what has been measured for alginate hydrogels. Last, adding to the cross-linking effect of Ca^2+^-ions, the entangled colloidal fibril network of TCNF can resist deformation effectively once the yielding starts. One suggested mechanism for this is the presence of contiguous structures, i.e., a continuous system of connections with “structural viscosity”, within the nanocellulose networks,^61^ which may also allow the formation of new Ca^2+^-carboxylate complexes even during deformation, creating a so-called “self-healing network” when TCNF transfer deformative load over the length of the fibrils.

The addition of PVA, which has previously been reported to act as a cross-linking agent in TCNF films,^41^ and suggested to aid in the cross-linking of TCNF hydrogels in our previous efforts,^11,34^ appears to have a net negative impact on the yield properties when compared to pure Ca^2+^-TNCF matrices. The TCNF-based matrices also had very weak mechanical properties without Ca^2+^-cross-linking, both with and without PVA. These results suggest that the ionic complexation between Ca^2+^ and TCNF is the primary cross-linking element in these matrices and that the TCNF-PVA cross-link would seem to require almost complete dewatering of the TCNF hydrogel for it to take place via esterification as reported earlier.^41^ The addition of anionic (alginate) or non-ionic (PVA) polymers into TCNF suspensions could induce entropic depletion-induced flocculating or adsorption and subsequent polymer bridging,^64^ but here, these effects seemed to be negated by the disruption of the contiguous TCNF network and ionic coordination.

Finally, we observed only a minor change in the wet mechanical properties of the matrices by the inclusion of entrapped *Synechocystis* cells. In our previous effort, we reported a decrease in yield properties for both TCNF- and alginate-based matrices using the same biomass loadings,^11^ but our larger dataset here seems to suggest that these changes are within the error margins. However, this is likely to change if higher biomass loadings are used and is a subject for future efforts.

### 3.2 Comparing the Porous Structures of Ca^2+^-Cross-Linked Matrices

**Figure 5:**
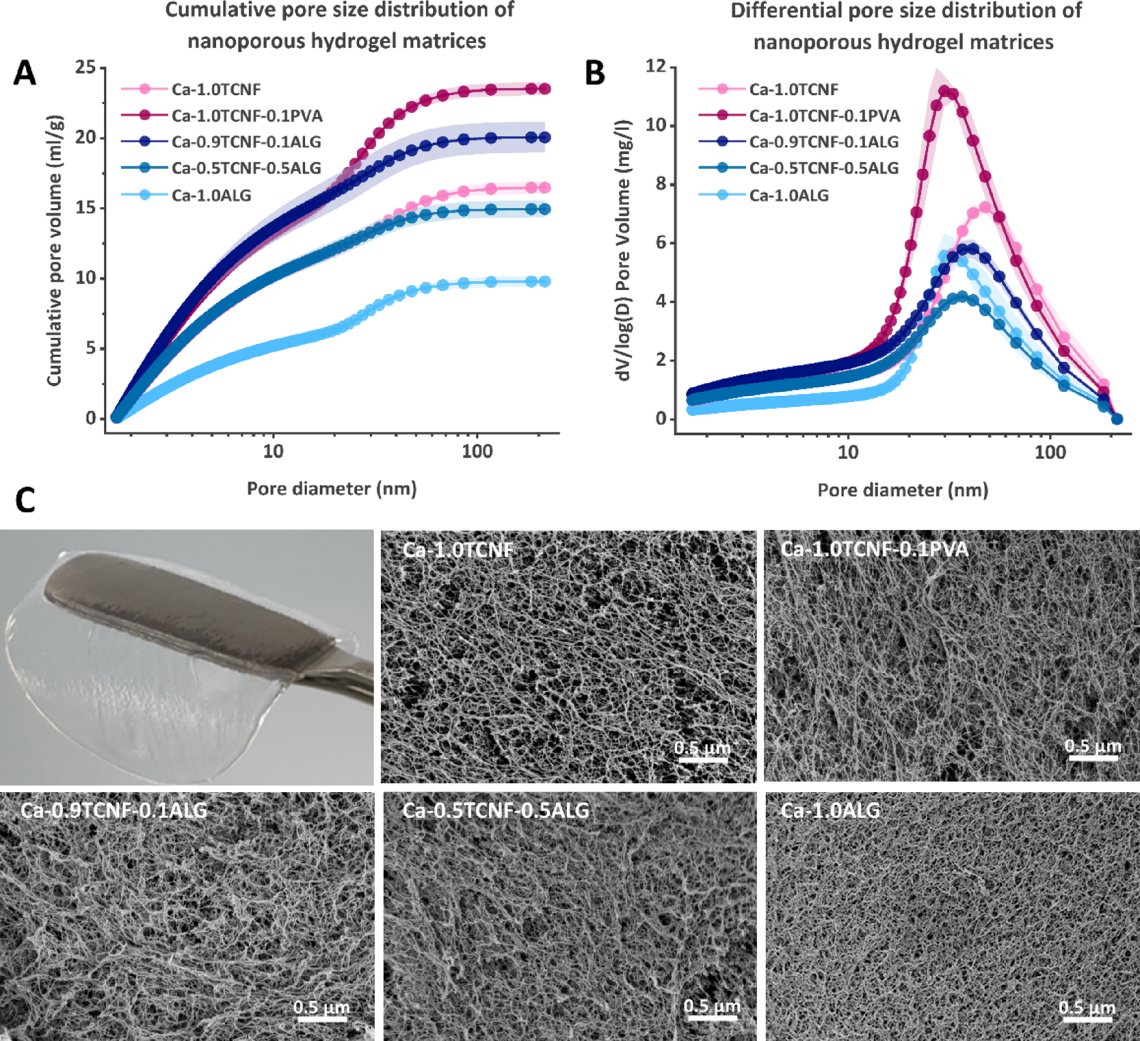
Porosity of the matrices. (A) Cumulative and (B) derivative pore size distribution of the water-swollen cross-linked matrices measured via TPM and (C) SEM images of the cross-linked hydrogel matrices prepared via critical point drying as well as a photo showing the appearance of a water-swollen Ca-1.0ALG hydrogel.

We used TPM to directly measure the mesoporosity and pore size distribution of the matrix hydrogels in the wet state. Figure 5A shows the cumulative and Figure 5B the differential pore size distribution (PSD) of the matrices after rinsing and swelling in water for over 48 h. In addition, we used SEM imaging of the same matrices (Figure 5C), prepared via critical point drying, to gain insight on the morphology and macroporosity of the materials.

As seen in Figure 5A, the cumulative pore volumes of the matrices increase in a heterogeneous and hierarchical manner, as indicated by the onset of a secondary slope at a pore size of approximately 20 nm. The largest total pore volume is observed for Ca-1.0TCNF-0.1PVA followed by 0.9TCNF-0.1ALG (Table 2), both of which have higher porosity than the other matrices, especially below the 20 nm pore size. Below them are Ca-1.0TCNF and Ca-0.5TCNF-0.5ALG with very similar pore volumes followed by 1.0ALG with a much lower pore volume than the other matrices. Thus, TCNF-based matrices were observed to have higher mesoporosity than pure Ca-alginate, but interestingly, a small addition of soluble PVA or alginate polymers to the TCNF network decreased its pore size but increased its total (meso)pore volume. In the SEM images (Figure 5C), all TCNF-containing matrices, including Ca-0.5TCNF-0.5ALG, seemed to share the typical appearance of a fibrillar network with larger pores than pure Ca-1.0ALG, which appears to have a somewhat denser network structure with much less apparent porosity. However, pure Ca-1.0TCNF seems to have the highest number of large pores based on the images. This can also be seen in the PSD in Figure 5B, where Ca-1.0TCNF peaks at the highest pore size at ∼50 nm and Ca-1.0ALG at the lowest at 30 nm. Ca-1.0TCNF-PVA also has a sharp peak at ca. 30 nm, and the peaks for Ca-0.9TCNF-0.1ALG and Ca-0.5TCNF-0.5ALG place in between the others at around 35 and 40 nm, respectively. The volume-weighed mean pore sizes shown in Table 2 correlate well with the PSD peaks, with the exception of Ca-1.0ALG, whose second highest mean pore size is likely due to its low pore volume at sizes below 20 nm.

**Table 2:** Solid Content, Volume Weighed Average Pore Size, and Total Pore Volume of the Swollen Matrices

matrix composition	solid content (wt %)	mean pore size (nm)	total pore volume (mL/g)
Ca-1.0TCNF	0.71 ± 0.010	52 ± 3.0	16 ± 0.44
Ca-1.0TCNF-PVA	0.70 ± 0.14	42 ± 0.44	24 ± 0.47
Ca-0.9TCNF-0.1ALG	0.71 ± 0.15	42 ± 0.12	20 ± 1.1
Ca-0.5TCNF-0.5ALG	0.84 ± 0.060	40 ± 0.47	15 ± 0.59
Ca-1.0ALG	0.91 ± 0.12	47 ± 2.6	9.8 ± 0.37

As expected, the water contents of the swollen matrices shown in Table 2 have increased from the original 99 wt % after matrix preparation. The primarily TCNF-based matrices have a water content of approximately 99.3 wt %, and the proportional addition of alginate decreases it moderately to approximately 99.1 wt % of Ca-1.0ALG, indicating a lower amount of swelling and thus lower overall porosity after the 48 h immersion in water.

Thermoporosimetry is used to characterize ridged mesoporous materials such as silica gels.^45^ It has also been successfully applied for swollen but largely insoluble materials such as cellulosic fibers, and the evidence to date is largely supporting the validity of this technique.^45^ Moreover, TPM also appears to give useful information for hydrated polymeric materials that are largely dissolved, such as alginate and PVA. However, for this material class, it is unclear if the usual Gibbs–Thomson coefficient is correct and if other effects such as ice crystal damage cause serious artifacts.

In the present study, we apply TPM to an even more challenging system: water-swollen complex multicomponent gels with colloidal TCNF and polymers that can be partially dissolved and partially cross-linked (PVA, alginate). The details of the water association in such systems are very complex and difficult to analyze. The organization of the polymers, localized steric effects, competitive hydration, and other effects is expected to affect the binding of water within the matrix. Nonetheless, the TPM results presented here seem to provide some insights on the porosity of the swollen gels. Another indication pointing toward the reliability of the measurement is that the total pore volume of Ca-1.0TCNF is very similar to that of a pure TCNF hydrogel with the same solid content measured using the same technique by Guccini et al.,^46^ which suggests that the results are reproducible.

The main finding from the TPM data was the difference in porosity between TCNF-containing and pure alginate matrices. This is likely due to their capacity to swell in water, which directly enlarges the cavities and loosens the matrix network. The high hygroscopicity of TCNF allows the network to hold more moisture and thus loosens the network and enlarges the porosity of the fibril network.^72^ Moreover, the different cross-linked structures could affect their morphology. Alginate polymers are softer than TCNF, but the egg-box structures of Ca^2+^-cross-linked alginate provide a relatively rigid network with some steric limitations in its orientations and much reduced electrostatic repulsion. This allows alginate to form tight and uniform mesoporous networks. On the other hand, Ca^2+^-TCNF cross-linking is sparser and allows the fibril network more freedom to swell, even if the TCNF network is rigid by itself.

The onset of a secondary slope in the cumulative PSD seen in all samples (Figure 5A) indicates the formation of hierarchical pore structures, as also discussed by Guccini et al.^46^ The largest heterogeneous behavior is observed with Ca-1.0TCNF-PVA, possibly due to PVA forming secondary structures inside the hydrogels. This is supported by the rheology measurements, indicating that TCNF and PVA are not directly attached to one another in the system. Similarly, the alginate in Ca-0.9TCNF-0.1ALG and Ca-0.5TCNF-0.5ALG could form heterogeneous networks with secondary structures, which would explain their relatively high pore volumes compared to pure Ca^2+^-cross-linked TCNF, while pure Ca^2+^-cross-linked alginate has a much less porous structure.

It is important to note that TPM is limited to detect the mesoporosity (2–50 nm) of a given material. Hence, we investigated SEM images of the same samples obtained via critical point drying (Figure 5C) to gain complementary insight on the morphology and macroporosity of the materials. This method avoids the crossing of phase transition boundaries, preventing the collapse of the fibrillar network more effectively than other SEM sample preparation methods such as air- or freeze-drying.^73,74^ Thus, even if the interactions between fibril and polymer chains after solvent exchange might not fully represent the interactions in the water-swollen matrix,^36,75,76^ qualitative insight of the matrix microstructure and macroporosity can be gained. The clearest observation from the images was that the cross-linked alginate polymers in Ca-1.0ALG create thin structures that form a more branched and more densely arranged network with no observed macroporosity, when compared to TCNF-containing matrices containing distinctly visible colloidal fibrils and much larger and more heterogeneously sized voids. This further highlights the distinctions between the two materials and their cross-linking pathways with Ca^2+^, supporting the main findings gained from both rheology and TPM measurements.

Otherwise, all TCNF-containing matrices seemed to possess a similar morphology with little visible distinctions besides a slightly higher amount of macropores on Ca-1.0 TCNF. Thus, the comparatively higher mesoporous volumes of Ca-1.0TCNF-0.1PVA and Ca-0.9TCNF-0.1ALG were not reflected in their apparent macroscale porosity. This could indicate that the suggested hierarchical structures within the colloidal TCNF network are more localized in nature. Indeed, their high pore volume especially at lower pore sizes below 20 nm (Figure 5A) combined with lower average pore size than pure TCNF matrices (Figure 5B) suggests that the soluble polymeric PVA and alginate can occupy the larger macropores otherwise filled with free water and create mesoporous structures within them, resulting in an increase in their mesoporous volume while the amount of visible macropores decreases. However, investigating these findings in more detail is a topic for future efforts. All in all, it appears that the SEM images are more in line with the PDS peaks in Figure 5B and the average pore sizes of the materials reported in Table 2 than the cumulative pore volumes shown in Figure 5A.

### 3.3 Investigation of Biocompatibility and Mass Transfer Properties of Matrices with Immobilized Cyanobacteria

**Figure 6:**
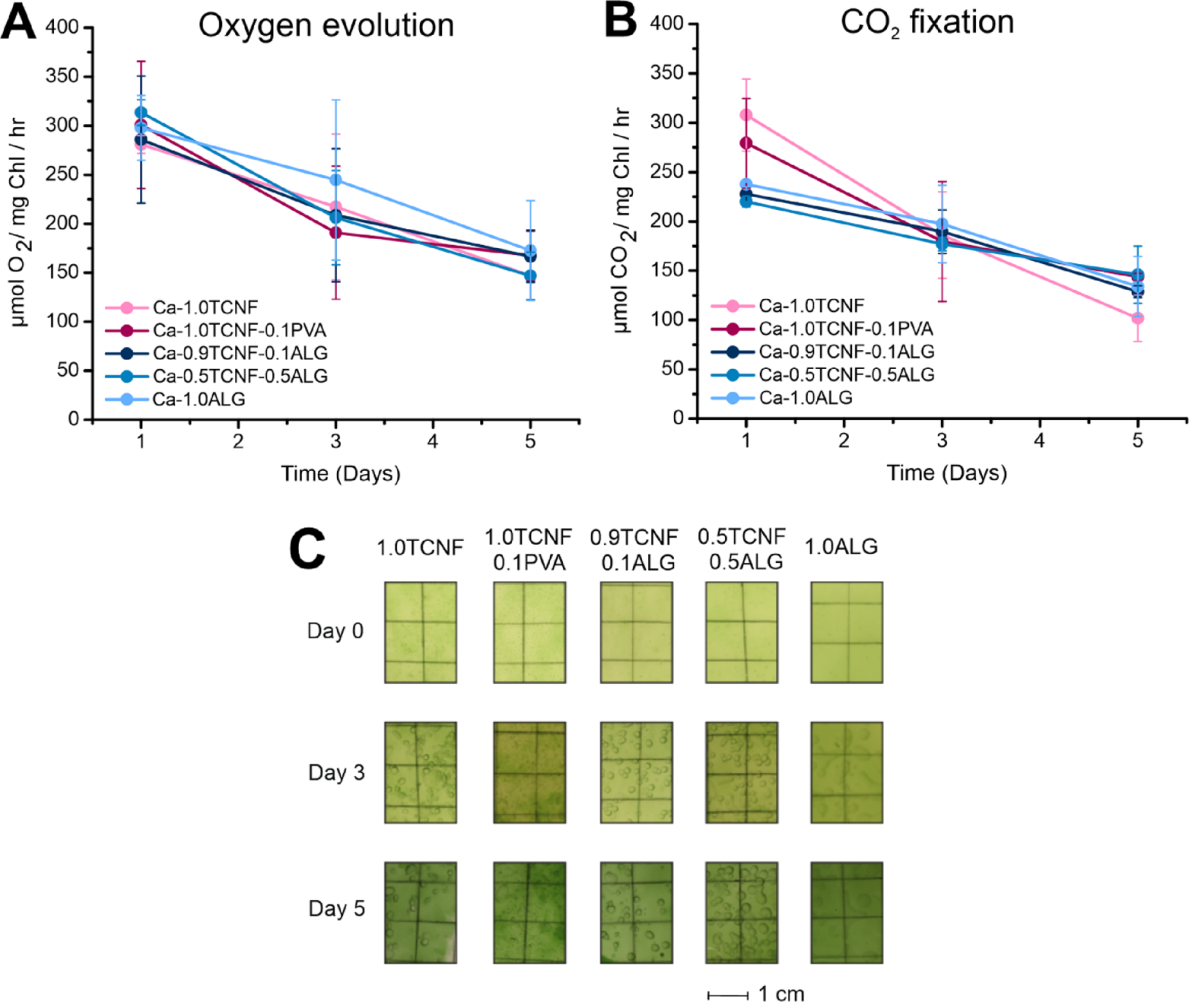
Rates of O_2_ evolution and CO_2_ uptake of cells entrapped in hydrogel matrices as measured by membrane inlet mass spectrometry. Maximal rates of net O_2_ evolution (A) and CO_2_ fixation (B) measured at the steady-state phase of photosynthetic induction calculated as average rates between 3 and 5 min after the onset of illumination. Values in panels (A, B) are means ± standard deviation from two (day 1) to three (days 3 and 5) biological replicates. (C) Photographs of the different hydrogel matrices with entrapped *Synechocystis* cells at 0, 3, and 5 days after entrapment.

We employed a highly sensitive MIMS approach to monitor O_2_ and CO_2_ gas fluxes of wild-type *Synechocystis* cells entrapped in the different hydrogel matrices for 5 min of illumination and in darkness to examine their photosynthetic electron transport and CO_2_ fixation capacities (Figure S5). Maximal rates of O_2_ evolution and CO_2_ uptake from the medium into the entrapped cells were reached after ca. 3–5 min of illumination when CO_2_ fixation reactions became activated. One day after cell immobilization, we measured high net O_2_ evolution and CO_2_ fixation rates, at ca. 300 μmol O_2_ mg Chl^–1^ h^–1^ and 225–300 μmol CO_2_ mg Chl^–1^ h^–1^, respectively, from all hydrogel matrix types (Figure 6A,B). Despite careful cell loading, fluctuations in the initial chlorophyll contents in the matrices magnified the variation in the measured gas fluxes. Still, we measured higher CO_2_ fixation rates from cells that immobilized Ca-1.0TCNF and Ca-1.0TCNF-0.1PVA hydrogel matrices in comparison to other matrix types (Figure 6B). Although these differences were not statistically significant according to a two-way analysis of variance (ANOVA), they seem to have a connection with the higher pore size and volume observed, respectively, in TCNF- and TCNF-PVA-based hydrogel matrices via thermoporosimetry and SEM imaging (Figure 5).

Three days after immobilization, the chlorophyll contents have stabilized to more equal values between matrix types, and we measured net O_2_ evolution and CO_2_ fixation rates around 200 μmol O_2_ mg Chl ^–1^ h^–1^ from all hydrogel matrices. No significant differences were detected between different matrices according to ANOVA (Figure 6A,B). Photosynthetic gas fluxes decreased further on day 5 after immobilization, reaching rates around 150 μmol O_2_ mg Chl^–1^ h^–1^ for net O_2_ evolution and CO_2_ fixation. The decrease in photosynthetic performance across all matrix types between day 1 and day 5 was statistically significant according to ANOVA (*p* < 0.05). Moreover, the most notable decrease was observed in the CO_2_ fixation capacity of cells immobilized in the Ca-1.0TCNF hydrogel matrix (Figure 6B). Photographs of the matrices (Figure 6C) indicate that all samples appeared relatively similar throughout the experiments, with slight differences in cell growth and accumulation of bubbles inside the matrices, which are most likely due to the heterogeneous pore structure of the samples. However, one noticeable difference was that Ca-1.0ALG showed less formation of macroscopic bubbles inside the matrix, which is in line with the observations from the SEM imaging that Ca-alginate has the tightest and most ordered pore structure with no observed macroporosity.

MIMS is a versatile method utilized to investigate in vivo exchange of gaseous compounds in cells or tissues, allowing real-time, simultaneous monitoring of, e.g., photosynthetic oxygen and carbon fluxes.^55^ Here, to our knowledge, a MIMS setup was used for the first time to study the O_2_ evolution and CO_2_ fixation in photosynthetic cells entrapped within thin hydrogel films. The MIMS measurement showed that both TCNF- and alginate-based immobilization matrices allow efficient evolution of O_2_ as well as uptake and fixation of CO_2_ (Figure 6 and Figure S5). Indeed, 3 days after immobilization, all the immobilized systems show gas exchange rates comparable to suspension cultures of *Synechocystis.*(77,78) However, the reduction in the rates through days 1–5 indicates that the exchange is hindered over time, either due to growth or biological changes in the immobilized cells or some restriction in the mass transfer properties of the matrices. The time-dependent differences between TCNF and other matrices also highlight that both the cells and the matrices are in a constant dynamic state in the SSPCF platforms.

Nonetheless, the results demonstrate that all immobilization strategies were conducive to CO_2_ uptake from the medium into the entrapped cells as well as the operation of the photosynthetic electron transport chain that allow efficient electron transport and carbon metabolism toward various photosynthesis-based products. This makes *Synechocystis* cells entrapped in all the self-standing hydrogel matrix compositions attractive platforms for various photosynthesis-based bioproduction applications, depending on which material and biological properties are most desirable.

When combined, our multidisciplinary analysis yielded some intriguing connections between photosynthetic performance and the distinct properties of the matrix materials. First, the larger intrinsic pore size and volume of TCNF-containing matrices in comparison to their alginate-based counterparts, as shown via TPM and SEM imaging in Section 3.2, likely allow more efficient photosynthetic CO_2_ exchange soon after immobilization. In contrast, the air bubbles that appear in TCNF-containing matrices after 3 and 5 days of cultivation (Figure 6C) could indicate impairment of photosynthetic gas exchange, as is also suggested by the notable decrease in maximum CO_2_ fixation rate 5 days after immobilization in the Ca-1.0TCNF matrix (Figure 6B). Our rheological analysis showed that the Ca-1.0TCNF hydrogel matrix had a contiguous structure with higher yield properties than other matrices (Figure 4B). Combined with the thixotropic behavior of TCNF, this may allow it to resist the loosening of the matrix network due cell division and the reversal of the ionic cross-linking due to ion exchange in the BG-11 medium^11^ more than the other matrices over time. By day 5, this may result in restricted gas exchange though the Ca-1.0TCNF matrix, limiting the cells’ ability to fix CO_2_ in the Calvin–Benson–Bassham cycle. In contrast, the initially more uniform and denser alginate matrix does not seem to have macroscopic bubble formation and maintains CO_2_ fixation performance slightly better than the TCNF matrix over time, suggesting that the high viscous behavior and low yield properties can facilitate the gas exchange, albeit at the cost of mechanical stability, due to higher tendency for swelling over time.

Further studies with larger sample sizes will be required for a more in-depth elaboration on the interdependencies between photosynthetic gas exchange and mass transfer or structural properties of the different hydrogel matrices. Nevertheless, this study provides a proof of concept demonstrating the applicability of gas flux analysis in the interdisciplinary development process of SSPCFs and providing means to study the photosynthetic performance and mass transfer limitations of the cells directly within immobilized systems. It also provides detailed information about the biocompatibility of the cells with their immobilization matrices and could aid in the estimation of changes in the biochemistry of the entrapped cells in comparison to cells cultivated in suspension. For example, combining MIMS measurements with absorbance spectrometry would produce highly specific data about the functionality of the whole photosynthetic electron transport chain as well as the downstream metabolic reactions of entrapped cells. Last, when combined with mechanical (rheology) and structural (TPM and SEM) analyses of the matrices, these biological performance indicators can be directly connected to the dynamic structural and mechanical properties of the matrices.

## 4 Conclusions

This study showcases an interdisciplinary toolbox of experimental methods that demonstrates how the evaluation of structure–property performance interfaces can assist in the development of TCNF- and alginate-based SSPCFs. The rheological data from the immobilization matrices without cells, interpreted with PCA, highlighted the importance of Ca^2+^-ions as the main cross-linking constituent. Moreover, matrices with a high TCNF concentration yielded at higher oscillation shear stresses (high σ_y_ and σ_c_) but possessed weaker rest behavior properties (*G*′, *G*″, and tan δ) than matrices with high alginate concentrations. These mechanical properties, which reflect the operational stability of the cell immobilization matrices, can be explained through the innate structural differences between the alginate polymer chains and colloidal contiguous TCNF “self-healing” network, as well as their ionic cross-linking mechanisms via egg-box coordination and Ca^2+^-carboxylate complexation, respectively. The results indicate that these properties can be controlled via altering, e.g., the G/M ratio and block lengths of alginate and the charge and fibril size of TCNF.

For the determination of porosity, we demonstrated that TPM can be utilized for complex water-swollen multicomponent gels with colloidal TCNF and partially dissolved and partially cross-linked polymers (alginate, PVA). The matrices form heterogeneous and hierarchical mesoporous networks, especially in the presence of non-cross-linked polymers (Ca-1.0 TCNF-PVA and Ca-0.9TCNF-0.1ALG). In general, TCNF-based matrices were more porous than the ones based on alginate. The difference in porosity is likely due to different swelling capacities between TCNF and alginate. SEM imaging supported the results from TPM in the macroporous range as Ca^2+^-cross-linked TCNF matrices appeared to form thicker networks with distinctly visible colloidal fibrils and large voids, while Ca^2+^-cross-linked alginate matrices formed visibly thinner and denser structures. Overall, the porosity measurements provide a link into the interface between the matrix structure and mass transfer properties.

The biological performance was studied with gas flux analysis by utilizing MIMS, for the first time, for cells entrapped within thin-layer hydrogel immobilization matrices. In general, gas flux analysis with MIMS unveils the operational status of the entrapped producer cells and, when combined with porosity measurements, can also be used to study mass transfer limitations across different immobilization systems. It was shown that all matrix compositions investigated here enabled efficient O_2_ evolution and CO_2_ fixation in entrapped photosynthetic cells, with levels comparable to suspension cultivations after 3 days of operation. Interestingly, we observed that a differential decrease in photosynthetic performance over time between hydrogel matrix types appeared to connect with the porosity and yield properties of the matrices. An interdisciplinary approach such as employed here consisting of the rheology, porosity, and gas exchange measurements can thus explain how the structure and mechanical properties are dynamically linked to the performance of immobilized photosynthetic cells. Moreover, our approach revealed the interdependency of properties such as porosity and mechanical stability: colloidal and thixotropic TCNF create a strong network during transition from rest to shear-induced deformation, and the TCNF-based matrices can resist the loss of ionic cross-linking well due to their contiguous and highly viscous nature. On the other hand, Ca-alginate matrices, which are initially rigid but may yield more easily, can offer improved gas exchange capabilities through additional matrix loosening over time. Overall, these techniques offer a way to interlink the mass transfer properties, porosity, and operational stability in the SSPCF development. Ultimately, the work shown here facilitates a pathway toward SSPCFs tailored to the specific needs of the production organism and process conditions.

## Supporting Information Available

Graphical overview of cell immobilization procedures; composition of the cell immobilization matrices before swelling; AFM images of Ca^2+^-cross-linked TCNF and alginate films; raw rheological data matrix used for PCA; scree plot for PC selection; rheological properties of self-standing matrices with and without *Synechocystis* cells; fluxes of O_2_ evolution and CO_2_ uptake in entrapped cells (PDF)
