# Supplementary material for: Mapping Nanocellulose- and Alginate-Based Photosynthetic Cell Factory Scaffolds: Interlinking Porosity, Wet Strength, and Gas Exchange
Source: Biomacromolecules. 2023 Jun 29;24(8):3484–97. doi: 10.1021/acs.biomac.3c00261 (PMC10428157; doi:10.1021/acs.biomac.3c00261)
Supplement: Supplementary file 1 — bm3c00261_si_001.pdf [file bm3c00261_si_001.pdf]

Mapping nanocellulose- and alginate-based photosynthetic cell factory scaffolds – interlinking porosity, wet strength and gas exchange

Tuukka Levä [1‡], Ville Rissanen [1‡,], Lauri Nikkanen [2‡], Vilja Siitonen [2], Maria Heilala [3], Josphat Phiri [4], Thaddeus C. Maloney [4], Sergey Kosourov [2], Yagut Allahverdiyeva [2], Mikko Mäkelä [1] and Tekla Tammelin [1,*]*

[1] VTT Technical Research Centre of Finland Ltd, VTT, PO Box 1000, FI-02044 Espoo, Finland

[2] Molecular Plant Biology, Department of Life Technologies, University of Turku, FI-20014 Turku, Finland

[3] Department of Applied Physics, Aalto University, FI-00076 Espoo, Finland

[4] Department of Bioproducts and Biosystems, Aalto University, FI-00076 Espoo, Finland

[‡]These authors contributed equally

* Corresponding authors: ville.rissanen@vtt.fi; Tekla.tammelin@vtt.fi

SUPPORTING INFORMATION

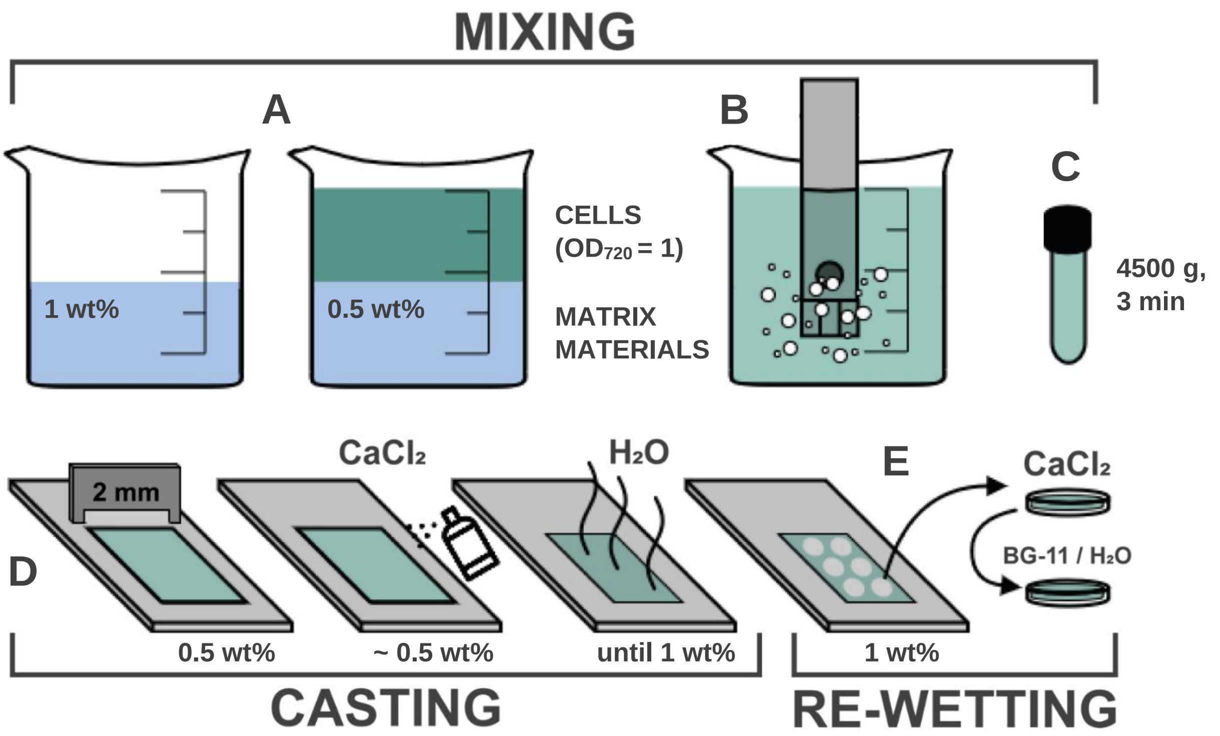

Figure S1. Graphical summary of the hydrogel matrix preparation steps for TCNF-based matrices. A) Mixing the matrix materials (total 1 wt%) and cells in BG-11 medium in 1:1 ratio, B) homogenization using Ultra-Turrax (12 000 rpm, 3 min), C) bubble removal via centrifugation (4500 g, 3 min), D) casting the hydrogel onto Teflon support, cross-linking by spraying 50 mM $CaCl_2$ and drying, E) swelling in 50 mM $CaCl_2$ for 15-30 min, then in BG-11 overnight before use. For alginate matrices without TCNF, drying step is excluded and 2 wt% alginate is mixed with cells to obtain final alginate concentration of 1 wt%.

Table S1. Composition of the hydrogel matrices before swelling in water (no cells) or BG-11 (with cells).

**Sample name**	**Ratio of matrix components**		**Cross-linker**		**Matrix solid content**	
	**TCNF**	**Alginate**	**$CaCl_2$ (Ca^{2+})**	**PVA (g)**	**Before drying**	**After drying**
Ca-1.0ALG	0 %	100 %	50 mM	-	1 wt% (no drying)	
Ca-0.5TCNF-0.5ALG	50 %	50 %	50 mM	-	0.5 wt%	1 wt%
Ca-0.9TCNF-0.1ALG	90 %	10 %	50 mM	-	0.5 wt%	1 wt%
Ca-1.0TCNF	100 %	0 %	50 mM	-	0.5 wt%	1 wt%
Ca-1.0TCNF-0.1PVA	100 %	0 %	50 mM	10 % of TCNF dry weight	0.55 wt%	1.1 wt%
1.0TCNF-0.1PVA	100 %	0 %	-	10 % of TCNF dry weight	0.55 wt%	1.1 wt%

1.0TCNF	100 %	0 %	-	-	0.5 wt%	1 wt%

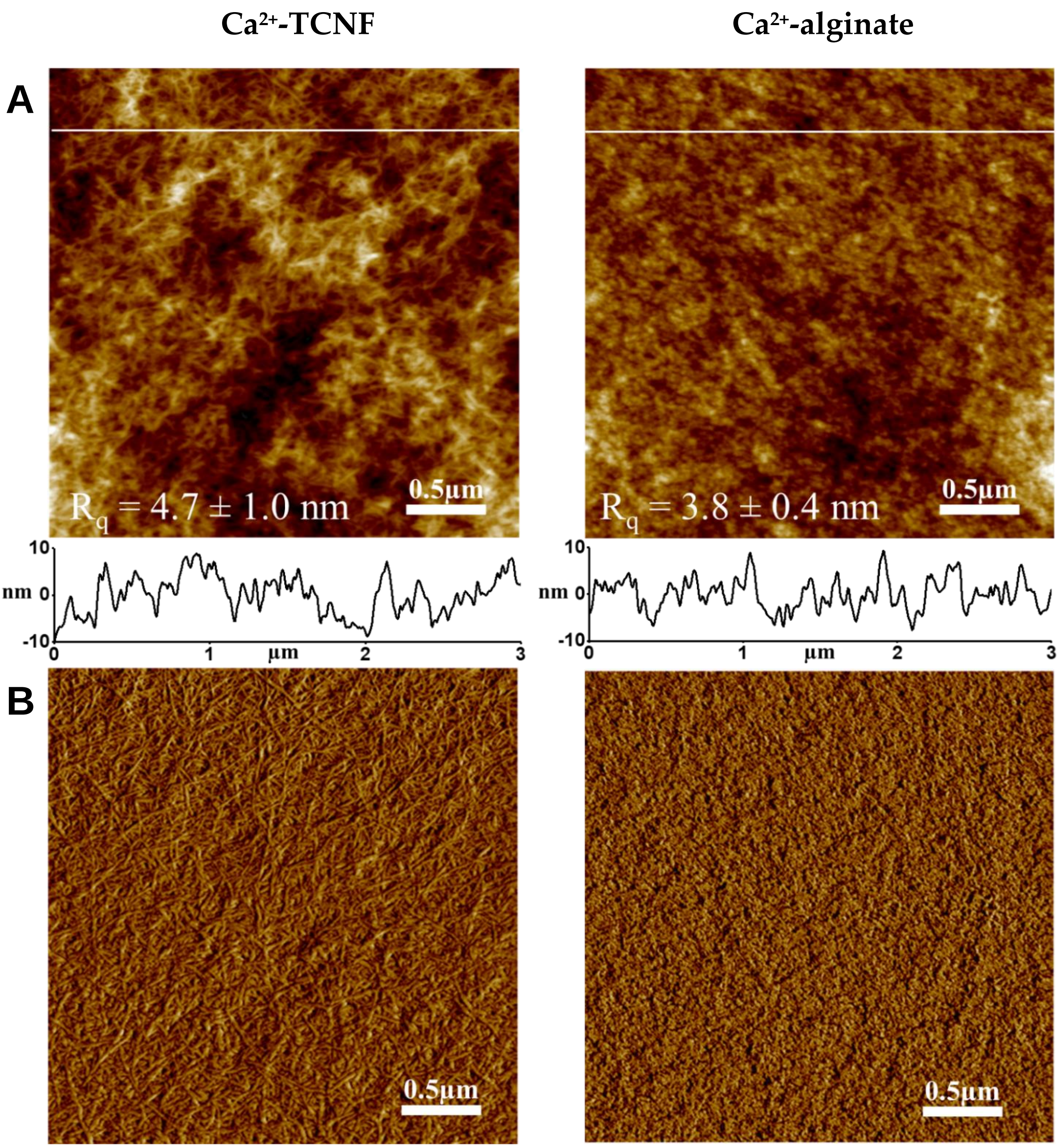

Figure S2. A) 3 × 3 µm AFM topography images of Ca^{2+}-cross-linked TCNF (left) and alginate (right) films, with height profile scans and average surface roughness (R_q) values, B) corresponding AFM phase contrast images.

Table S2. Raw rheological data used in the PCA.

Sample	G' (Pa)	G'' (Pa)	tan δ	σ_y (Pa)	σ_c (Pa)
Ca-1.0ALG	3698.12	420.346	0.11367	5.00637	50.0467
	3004.86	338.455	0.11264	15.8276	79.3027
	1886.25	233.074	0.12357	6.30151	19.9235
	3170.71	336.192	0.10603	7.93503	50.0485
	1661.19	204.866	0.12333	12.5441	49.902
	2671.97	389.769	0.14587	6.30371	19.9309
	1967.98	249.234	0.12664	9.98334	50.0227
Ca-0.5TCNF-0.5ALG	1955.6	198.686	0.1016	50.0468	79.3121
	2010.1	200.955	0.09997	31.5661	62.9718
	1902.26	192.12	0.101	31.5891	63.0197
	948.177	103.777	0.10945	19.903	62.9137
	747.75	81.7566	0.10934	15.7811	31.4728
Ca-0.9TCNF-0.1ALG	1088.7	83.9755	0.07713	15.7789	39.6084
	1591.64	118.501	0.07445	62.9378	99.7303
	996.799	70.8137	0.07104	25.0191	49.9003
	1374.96	107.35	0.07808	31.548	62.9326
	1620.62	130.806	0.08071	62.9855	125.648
	1277.38	114.719	0.08981	39.719	79.2292
Ca-1.0TCNF	982.625	65.4914	0.06665	15.7791	39.6044
	1807.08	101.302	0.05606	49.9549	99.6335
	2202.97	132.674	0.06023	62.9227	157.988
	1372.34	77.77	0.05667	25.0095	49.8764
	1972.65	122.112	0.0619	49.9768	157.969
	1922.74	113.711	0.05914	79.1915	157.957
	1681.6	110.829	0.06591	31.5255	99.6508
	1606.17	102.714	0.06395	49.9582	157.907
	1861.72	115.977	0.0623	62.9215	157.992
Ca-1.0TCNF-0.1PVA	1645.6	108.996	0.06623	25.06	71.06526
	1443.37	93.4507	0.06474	25.0461	62.8887
	1543.54	106.32	0.06888	62.9311	102.88115
	1559.88	107.314	0.0688	39.7067	99.6982
	1840.55	120.88	0.06568	62.9659	158.113
	1483.5	101.681	0.06854	49.9995	125.556
	1280.58	90.7615	0.07088	50.0091	99.757
	1587.7	102.883	0.0648	49.9936	125.535
	1606.54	95.4009	0.05938	62.9331	125.529
1.0TCNF-0.1PVA	15.2599	1.95869	0.12836	1.74612	6.37168
	14.1485	1.54428	0.10915	1.41412	4.24136
	21.6926	2.09724	0.09668	2.31674	8.68388
	22.2758	2.0985	0.09421	2.42759	7.32966
	25.128	1.99854	0.07953	3.2163	12.163
	15.737	1.70085	0.10808	1.76899	8.16492
1.0TCNF	15.0304	1.89808	0.12628	1.7986	2.79481
	13.1179	1.64268	0.12522	1.33284	3.20087
	14.1778	1.823	0.12858	1.718	4.11414
	18.7144	2.0692	0.11057	1.94004	5.84427

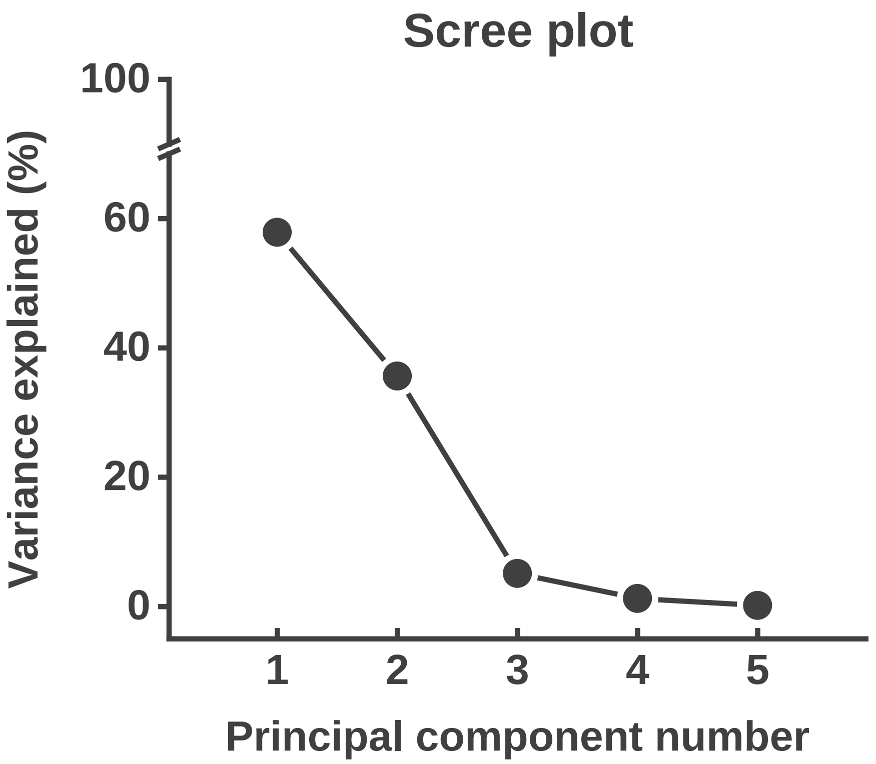

Figure S3. Scree plot used to justify the selection of PCs from the PCA performed on normalized and mean-centered data with unit variance.

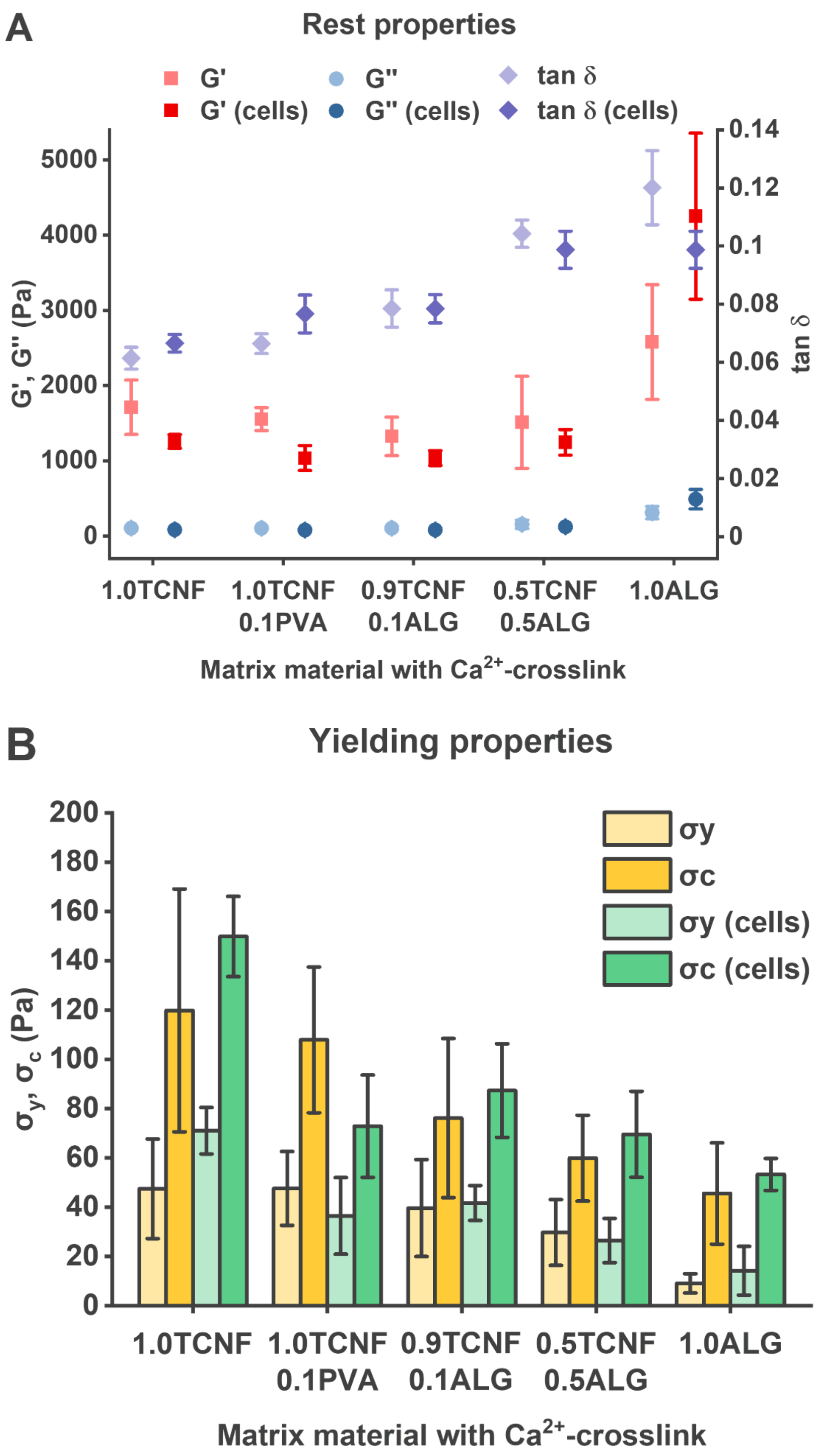

Figure S4. Rheological properties of hydrogel matrices with and without *Synechocystis* wild-type cells. A) Rest properties (G', G'', Tan δ), and B) yielding properties, i.e. yield stress (σ_y) and critical stress (σ_c).

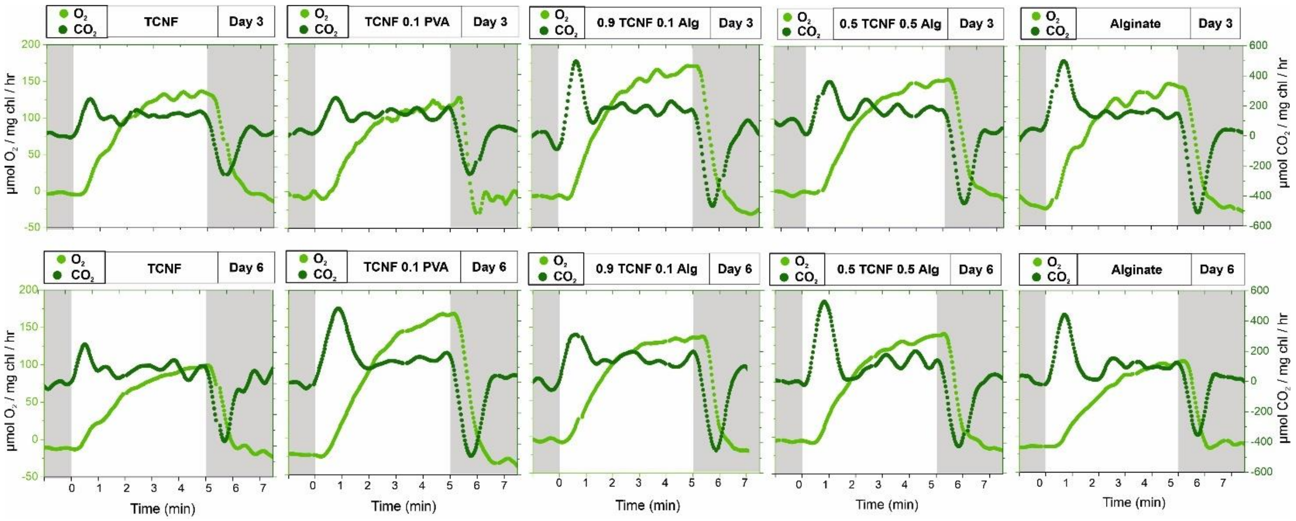

Figure S5. O_2 and CO_2 fluxes of *Synechocystis* wild-type cells entrapped in hydrogel matrices as measured by membrane inlet mass spectrometry. Rates of gross O_2 evolution and CO_2 uptake before, during, and after 5 min illumination at 500 µmol photons m^{-2} s^{-1}., as measured 3 and 5 days after cell entrapment within hydrogel matrices of different compositions. Entrapped cells were dark-adapted for 5 min before illumination.
